# Elective staged proctocolectomy and living donor liver transplantation for colon cancer with sclerosing cholangitis-related ulcerative colitis: a case report

**DOI:** 10.1186/s40792-020-01059-6

**Published:** 2020-11-01

**Authors:** Yoshihiro Miyagi, Tatsuya Kinjo, Tomoharu Yoshizumi, Noboru Harada, Shingo Arakaki, Tetsu Kinjo, Akira Hokama, Mitsuhisa Takatsuki

**Affiliations:** 1 Department of Digestive and General Surgery, Graduate School of Medicine, University of the Ryukyus, 207 Uehara, Nishihara, Okinawa 903-0125 Japan; 2 Department of Surgery and Science, Graduate School of Medical Sciences, Kyushu University, Fukuoka, Japan; 3 Department of Infectious, Respiratory, and Digestive Medicine, University of the Ryukyus, Okinawa, Japan

**Keywords:** Colitis-related colon cancer, Ileal pouch–anal anastomosis, Inflammatory bowel disease, Small-for-size syndrome

## Abstract

**Background:**

Primary sclerosing cholangitis (PSC) is a well-known complication of ulcerative colitis (UC), but it is rare to encounter patients requiring both living donor liver transplantation (LDLT) and proctocolectomy. We report a case of elective two-stage surgery involving proctocolectomy performed after LDLT for a patient with early colon cancer concurrent with PSC-related UC. To our knowledge, this is the first report of concurrent cancer successfully treated with both LDLT and proctocolectomy.

**Case presentation:**

A 32-year-old Japanese man with colon cancer associated with UC underwent restorative proctocolectomy at 3 months after living donor liver transplantation (LDLT) for PSC. He was diagnosed with PSC and UC when he was a teenager. Conservative therapy was initiated to treat both PSC and UC. He had experienced recurrent cholangitis for years; therefore, a biliary stent was placed endoscopically. However, his liver function progressively deteriorated. Colonoscopic surveillance revealed early colon cancer; hence, surgical treatment was considered. PSC progressed to cirrhosis and portal hypertension; hence, LDLT was performed before restorative proctocolectomy. Three months after LDLT, we performed restorative proctocolectomy with ileal pouch–anal anastomosis. The postoperative course was uneventful. The patient was well, with good liver and bowel functions and without tumor recurrence, more than 1 year after proctocolectomy.

**Conclusions:**

With strict patient selection and careful patient management and follow-up, elective proctocolectomy may be performed safely and effectively after LDLT for concurrent early colon cancer with PSC-related UC. There are no previous reports of the use of both LDLT and proctocolectomy for the successful treatment of PSC-related UC and concurrent cancer.

## Background

Primary sclerosing cholangitis (PSC) is one of the most common indications for liver transplantation (LT) and is a well-known complication of ulcerative colitis (UC) because of the associated autoimmune characteristics [1–4]. UC is a well-recognized risk factor for colon cancer [5–8]; therefore, we occasionally encounter a complicated situation in which a patient with PSC develops concurrent UC-related colon cancer. When the liver function is adequate, isolated proctocolectomy might be reasonable. However, if liver failure becomes deteriorated enough to require LDLT, then it is difficult to decide which treatment procedure to use because of the difficulty in performing isolated proctocolectomy. We report a case of elective two-stage surgery involving proctocolectomy after LDLT for concurrent early colon cancer with PSC-related UC.

## Case presentation

**Fig. 1:**
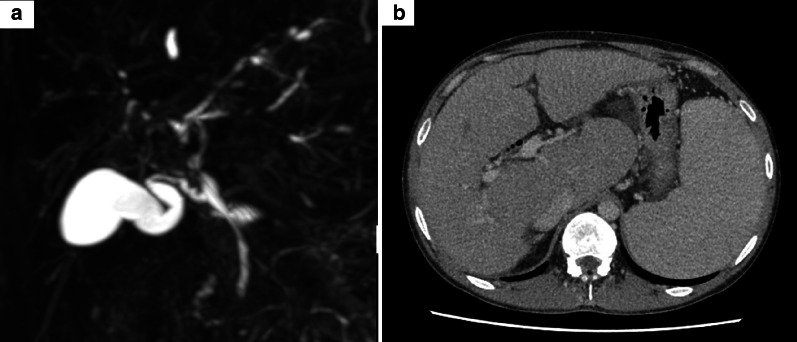
The imaging findings of primary sclerosing cholangitis (PSC) are shown. **a** Magnetic resonance cholangiopancreatography revealed typical features of PSC with irregular narrowing of bile ducts, stenoses, and focal dilatation of bile ducts. **b** Computed tomography revealed a cirrhotic liver with an enlarged caudate lobe and splenomegaly

**Fig. 2:**
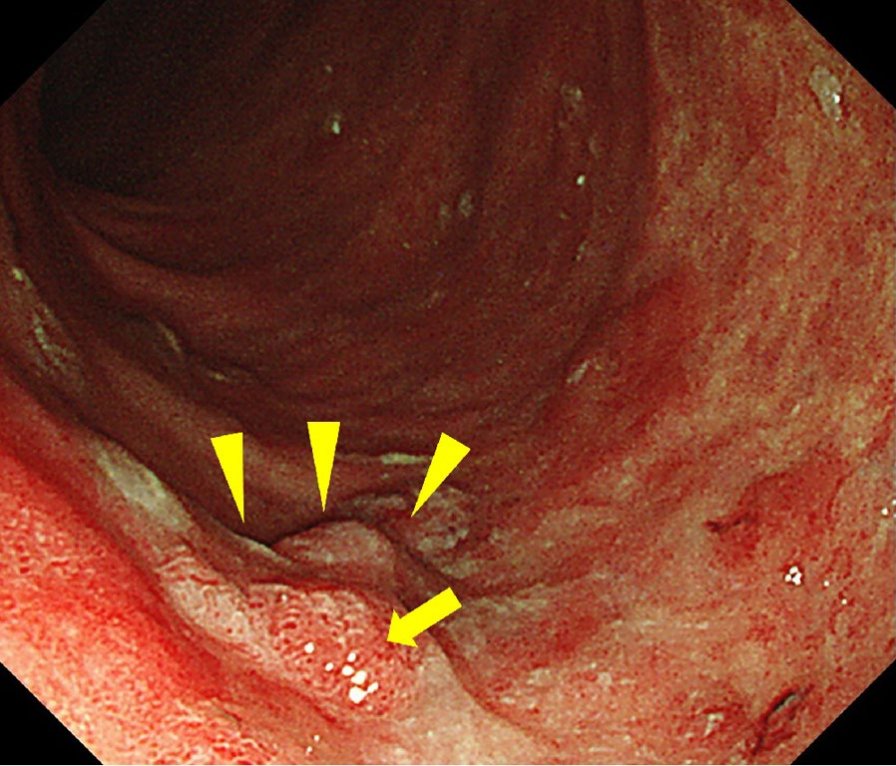
Surveillance colonoscopy image showing an elevated lesion in the ascending colon (arrowheads). This lesion was diagnosed as well-differentiated adenocarcinoma based on the biopsy findings (arrow shows the biopsy area)

A 32-year-old man was admitted to our hospital to undergo surgery for colon cancer associated with pancolitis-type UC that had been diagnosed 20 years previously. He was simultaneously diagnosed with PSC (Fig. 1); therefore, his UC was considered PSC-related. PSC and UC had been well-controlled until the development of severe colitis, diagnosed as toxic megacolon 5 years previously and treated using aminosalicylates, azathioprine, and adalimumab; cholangitis was also present, which was treated with endoscopic biliary stenting. After colitis and cholangitis were treated, the patient had been well, and his liver function remained acceptable. However, his total bilirubin level gradually increased to approximately 5–8 mg/dl and colon cancer developed. Surveillance colonoscopy revealed two superficial neoplastic lesions. Slightly elevated lesions were detected in the ascending and transverse colon (Fig. 2). Pathological biopsy findings indicated well-differentiated tubular adenocarcinoma in the ascending colon and an adenoma in the transverse colon. Because mucosal reddening was observed in the colon (Mayo endoscopic subscore 1–2), we diagnosed UC-related colon cancer. Isolated proctocolectomy was initially considered, but the patient’s liver function progressively deteriorated with severe concurrent portal hypertension. The Child–Pugh classification was C (score 11), and the Model for End-Stage Liver Disease score was 15. Because his UC-related colon cancer was diagnosed as T1bN0M0 stage I, we expected a postoperative outcome similar to that of sporadic colon cancer [9–11], which has an estimated 5-year survival rate of > 90% according to the Japanese Society for Cancer of the Colon and Rectum sporadic cancer data [12]. Therefore, we eventually decided to perform LDLT before restorative proctocolectomy.

**Fig. 3:**
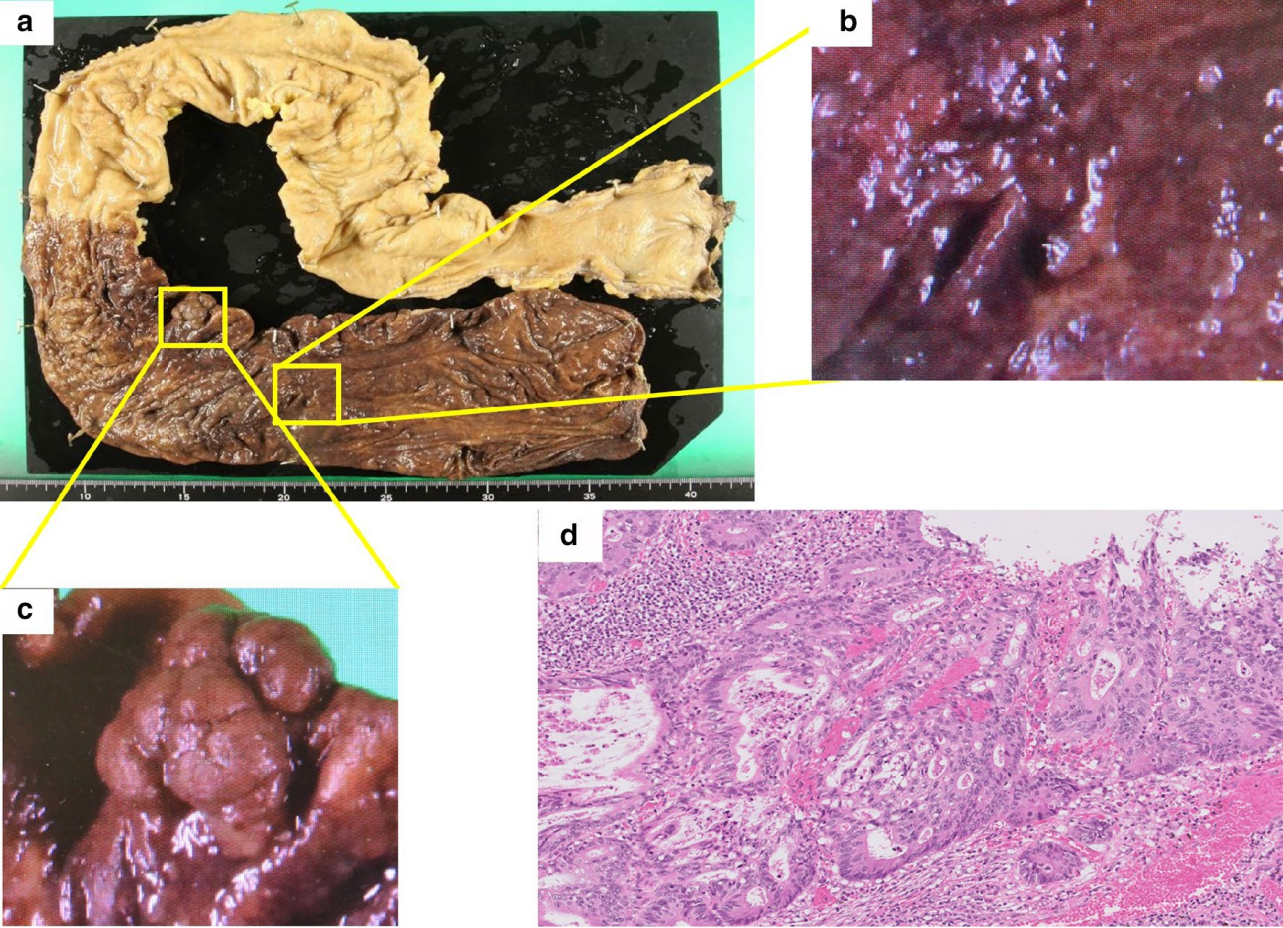
**a** Macroscopic findings of the resected lesion. Severe inflammation was observed in the whole colonic mucosa and the right colon was brown because of mucosal hemorrhages. **b** Slightly elevated lesion in the ascending colon. **c** Slightly elevated lesion in the transverse colon. **d** Histological examination of the tumor revealed well-differentiated to moderately differentiated adenocarcinoma in the mucosa of the ascending colon (hematoxylin and eosin staining; magnification, ×200)

**Fig. 4:**
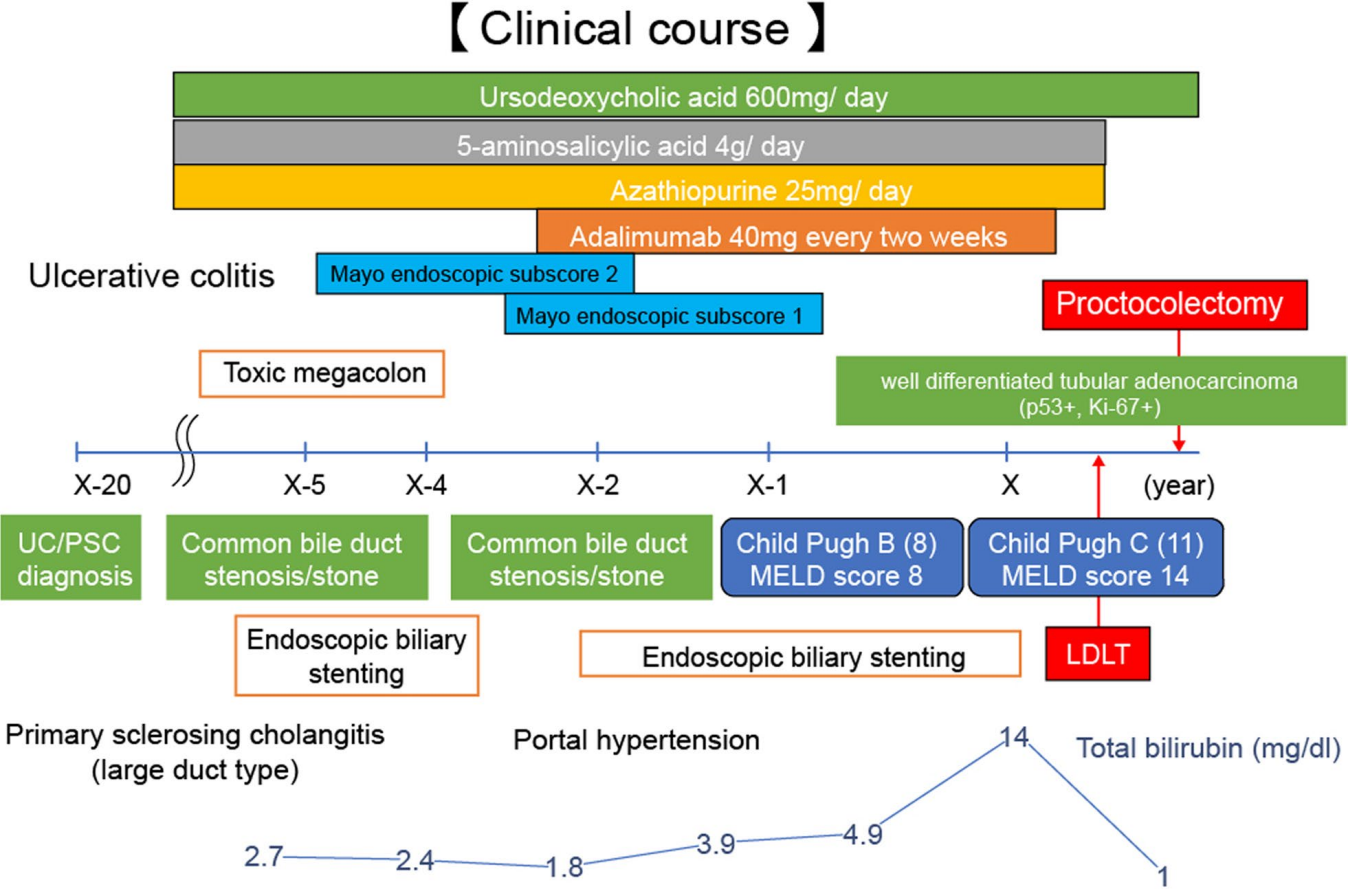
Clinical course of the patient since PSC and UC were diagnosed

LDLT for PSC was performed using a relatively small-for-size (463 g; ratio of the graft volume to the recipient standard liver volume, 38.9%) right liver graft from his 27-year-old wife. Because their blood types were incompatible, rituximab was administered for 2 weeks before surgery. During surgery, severe adhesion was observed between the right lobe and diaphragm with multiple collateral veins that contributed to a large amount of bleeding; however, LDLT was successfully performed without any other significant complications. Biliary reconstruction using Roux-Y hepaticojejunostomy (instead of duct-to-duct reconstruction) was performed, as generally adopted for LT for PSC. The operative time was 619 min and the estimated blood loss was 16,761 g. The postoperative course was uneventful except for the development of small-for-size syndrome with massive ascites (1120 ml/day). The peak total bilirubin level was 20 mg/dl at 10 days after surgery. The patient was discharged on postoperative day 20 after his liver function had normalized. Basic immunosuppression medication consisted of tacrolimus, steroids, and mycophenolate mofetil. There were no signs of rejection. Follow-up colonoscopy was performed at 1 month after LDLT, and no exacerbation of colon cancer was detected. Three months after LDLT, we performed restorative proctocolectomy with ileal pouch–anal anastomosis. Severe adhesion was observed in the upper abdomen. After resection of the colon, a J-pouch was constructed and returned to the abdomen. A hand-sewn ileal pouch–anal anastomosis was created and a temporary diverting ileostomy was constructed. The operative time was 572 min and the estimated blood loss was 1445 g. The postoperative course was uneventful, and the patient was discharged on postoperative day 16. Severe inflammation was observed in the whole resected colonic mucosa, and the right colon was brown because of mucosal hemorrhages (Fig. 3a). Two superficial neoplastic lesions were detected in the ascending and transverse colon. Pathological examination revealed well-differentiated to moderately differentiated adenocarcinoma in the mucosa of the ascending colon without lymph node metastasis (Fig. 3b, d). A low-grade tubular adenoma was observed in the transverse colon (Fig. 3c). Infiltration of inflammatory cells to the submucosa was observed in the colon. After surgical dilatation of the anastomosis stricture, ileostomy reversal was performed 10 months after proctocolectomy. The patient was well thereafter, with good liver and bowel functions and without tumor recurrence, at > 1 year after proctocolectomy. The entire clinical course of this case is shown in Fig. 4

## Discussion

UC is a well-recognized complication of PSC. The current comorbidity rate of UC for patients with PSC in Japan ranges from approximately 20% to 55%, which is lower than the range of 30% to 80% for Western cohorts [1–4]. Although UC is also recognized as a risk factor for the development of dysplasia or cancer [5–8], we rarely encounter a patient with PSC-related UC and concurrent colon cancer requiring both LT and colectomy, as in the current case. Fukuhara et al. reported a case of proctocolectomy for colon cancer with PSC-related UC that was detected after LDLT [13]. However, there are no reports of concurrent cancer successfully treated with both LDLT and proctocolectomy.

The treatment of this case presented several challenges. The first involved the timing of proctocolectomy. Proctocolectomy could not be independently performed without LT because of severe liver failure. Simultaneous proctocolectomy with LDLT without a stoma could have resulted in anastomotic leakage as a potentially fatal complication. Even with the stoma, we were concerned that it might be difficult to control the fluid balance due to the possible massive fluid loss, not only from the ascites, but also from the intestine. Poritz et al. reported that patients with PSC who require colectomy can undergo simultaneous orthotopic LT and total abdominal colectomy; thereafter, they can be candidates for subsequent ileal pouch–anal anastomosis reconstruction when the liver function has improved [14]. However, unlike whole LT, small-for-size syndrome with massive ascites is a specific and significant complication of LDLT [15]. The patient developed small-for-size syndrome with a peak total bilirubin level of 20 mg/dl and massive ascites. However, if we had performed LDLT first without proctocolectomy, then bacterial translocation might have been a concern, especially with immunosuppression, because inflammatory bowel diseases, including UC, are known to cause a high risk of bacterial translocation [16–19]. Eventually, we planned to perform two-stage surgery with LDLT first because UC was expected to be controlled with immunosuppression. Fortunately, the posttransplant course was uneventful, and we were able to safely perform proctocolectomy after the patient had fully recovered at 3 months after LDLT.

The second challenge was the indication of LT with concurrent nonhepatic malignancy. Kosai-Fujimoto et al. reported that there was no significant difference in survival after LDLT for patients with and without nonhepatic malignancy, including nine concurrent cases [20]. They concluded that elective resection was successful if the tumors were at an exceedingly early stage. The UC-associated colorectal cancer patients had a prognosis that was similar to that of sporadic colorectal cancer at an early stage [9–11]. In our case, colon cancer was pathologically diagnosed as TisN0M0 stage 0; therefore, the prognosis was expected to be similar to that of sporadic colon cancer, which has a chance of recurrence-free survival without additional treatment of > 90% according to the Japanese Society for Cancer of the Colon and Rectum [12]. With UC, the incidence of poorly differentiated cancer with multiple developments is reportedly higher than that of cases without UC; this is one of the reasons why proctocolectomy is recommended [9–11, 13]. In the current case, because we did not find any evidence of poor differentiation characteristics in the colon tumor, we performed careful patient follow-up with minimal immunosuppression and planned to perform proctocolectomy as soon as possible after LDLT. We selected ileal pouch–anal anastomosis, which has been proven to be a safe approach after LT, as the bowel resection procedure [14].

PSC recurrence after LT, which contributes to poor patient and graft survival, especially after LDLT, has been well-recognized. Egawa et al. showed that the risk of re-transplantation for graft loss due to recurrence of PSC after LDLT is high, and that the risk factors for recurrence are high MELD scores (> 24), first-degree relative donors, postoperative cytomegalovirus infection, and early biliary anastomotic complications [21]. These did not occur in this case. Concomitant IBD was not associated with recurrence in their study [21]. We have not observed any signs of PSC recurrence in this case; however, regular liver biopsies and careful follow-up must be performed to monitor for recurrence.

## Conclusions

With strict patient selection and careful patient management and follow-up, elective proctocolectomy may be a safe and effective procedure after LDLT for concurrent early colon cancer with PSC-related UC.

## Data Availability

The authors declare that all of the data in this article are available within the article.

## Abbreviations

LDLT: Living donor liver transplantation
LT: Liver transplantation
PSC: Primary sclerosing cholangitis
UC: Ulcerative colitis
